# Ability to Generate Patient Registries Among Practices With and Without Electronic Health Records

**DOI:** 10.2196/jmir.1166

**Published:** 2009-08-10

**Authors:** Adam Wright, Elizabeth A McGlinchey, Eric G Poon, Chelsea A Jenter, David W Bates, Steven R Simon

**Affiliations:** ^4^Department of Ambulatory Care and PreventionHarvard Medical School and Harvard Pilgrim Health CareBoston, MAUSA; ^3^Clinical and Quality AnalysisPartners Healthcare SystemBoston, MAUSA; ^2^Division of General MedicineBrigham & Women’s HospitalBoston, MAUSA; ^1^Clinical Informatics Research & DevelopmentPartners Healthcare SystemBoston, MAUSA

**Keywords:** Registries, chronic disease, quality assurance, health care, primary health care, family practice, hospital information systems, medical records systems, computerized

## Abstract

**Background:**

The ability to generate registries of patients with particular clinical attributes, such as diagnoses or medications taken, is central to measuring and improving the quality of health care. However, it is not known how many providers have the ability to generate such registries.

**Objectives:**

To assess the proportion of physician practices that can construct registries of patients with specific diagnoses, laboratory results, or medications, and to determine the relationship between electronic health record (EHR) usage and the ability to perform registry functions.

**Methods:**

We conducted a mail survey of a stratified random sample of physician practices in Massachusetts in the northeastern United States (N = 1884). The survey included questions about the physicians’ ability to generate diagnosis, laboratory result, and medication registries; the presence of EHR; and usage of specific EHR features.

**Results:**

The response rate was 71% (1345/1884). Overall, 79.8% of physician practices reported being able to generate registries of patients by diagnosis; 56.1% by laboratory result; and 55.8% by medication usage. In logistic regression analyses, adjusting for urban/rural location, practice size and ownership, teaching status, hospital affiliation, and specialty, physician practices with an EHR were more likely to be able to construct diagnosis registries (adjusted odds ratio [OR] 1.53, 95% confidence interval [CI] 1.25 - 1.86), laboratory registries (OR 1.42, 95% CI 1.22 - 1.66), and medication registries (OR 2.30, 95% CI 1.96 - 2.70).

**Conclusions:**

Many physician practices were able to generate registries, but this capability is far from universal. Adoption of EHRs appears to be a useful step toward this end, and practices with EHRs are considerably more likely to be able to carry out registry functions. Because practices need registries to perform broad-based quality improvement, they should consider adopting EHRs that have built-in registry functionality.

## Introduction

With the publication of the Institute of Medicine’s *Crossing the Quality Chasm* report [1], physicians and health care delivery systems have sharpened their focus on the care of populations and panels of patients. Managing the health of populations demands the ability to identify individuals within the population with specific clinical or sociodemographic characteristics. The core of population management is the patient registry—a list of multiple patients who share some common clinical trait, such as being overdue for a laboratory test, receiving a medication, or having a particular diagnosis.

Registries play a key part in the Chronic Care Model [2,3], a broadly promulgated framework for managing patients with chronic illnesses. In the traditional paradigm of care, a physician reviews the record of, and evaluates, one patient at a time. In the paradigm of the Chronic Care Model and population health management, the physician looks simultaneously at all of his or her patients with a particular diagnosis or combination of diagnoses. This activity is commonly, but not universally, carried out by utilizing an electronic health record (EHR) [4,5].

Laboratory result registries have been used for several purposes, but the most common is detection of patients overdue for screening tests [6-8]. Laboratory result registries can also serve as surrogates for, or adjuncts to, diagnosis-based registries [9,10]. In comparison, medication registries can be used to identify and communicate with patients who are receiving a medication for which some change is recommended or required, as in the case of a safety recall or the new availability of a more effective or less expensive alternative [11-13].

Taken together, this evidence suggests that diagnosis, medication and laboratory registries are essential and effective tools for improving the quality and safety of health care at the population level. A variety of studies at different sites, using different registries and with different disease foci have shown positive results [2-3,6-13]. It is not known, however, how easy or difficult it is for practices in the community to generate such registries. Furthermore, it is apparent that many studies demonstrating the potential for registries to improve care arise in settings with robust electronic health records and computerized provider order entry systems [4,6,8,11,12].

In order to understand better the registry generation capabilities of community ambulatory practices, as well as the relationship between EHR usage and registry capabilities, we undertook the present study. Our goal was to measure physicians’ general abilities to perform registry functions in office practice and to explore further the hypothesis that use of electronic health records is associated with the ability to perform registry functions. This study is one aim of a larger study which used a variety of methods, including surveys, focus groups, direct observation, and quality assessment. The goal of the larger study was to measure adoption and use of EHRs in Massachusetts and to compare state-wide adoption to three specific communities in the state that were in the process of implementing community-wide electronic health records with information exchange. Other results of the larger study have been have published previously [14,15].

## Methods

### Sampling

We carried out a statewide survey of physician practices in Massachusetts between June 2005 and November 2005. We began with a commercial database of physicians in Massachusetts (Folio Associates, Hyannis, MA) which contained contact information for 20,704 physicians practicing at 6308 distinct practice sites in the state. We drew a stratified random sample of practice sites from this database and selected one physician at random from each practice. Our sample was stratified by geography (urban vs nonurban based on county designation, except in the case where there were rural ZIP codes in urban counties, where ZIP codes were used instead), practice size (1 physician, 2 - 3 physicians, 4 - 6 physicians, and 7 or more physicians, exclusive of residents), and practice type (hospital-based primary care, hospital-based specialty/mixed, non-hospital-based primary care, or non-hospital-based specialty/mixed). These sampling characteristics were based on values in the commercial database.

Practices in rural parts of Massachusetts, primary care practices within hospitals, and large practices were oversampled by 100% in our sampling plan to ensure we had adequate representation of these particular practice types. Further details of the sampling plan have been reported previously [14,15].

### Survey

Ultimately, we identified a sample of 1884 physician practices across the state of Massachusetts. We mailed a randomly chosen physician at each practice the survey and a US $20 cash incentive to encourage participation. We contacted non-respondents by phone several times and also sent the survey to them two more times (without further cash incentive). The survey contained demographic questions (relating to practice size, teaching, and practice ownership), as well as a variety of questions about quality improvement, practice satisfaction, use of technology, and finances. Some of these questions have been analyzed as part of other aims of this study [14,15]. Two questions (practice size and type) were asked in the survey and were also present in the commercial database used for sampling. In the case that a practice’s response differed from the commercial database (eg, because the practice had changed in size), the survey responses were used in analysis as they were more current.

The survey also asked questions about the use of registry functions and availability of an EHR (defined as “an integrated clinical information system that tracks patient health data, and may include such functions as visit notes, prescriptions, lab orders, etc”) in the physician’s practice. Specifically, physicians were asked to rate the ease of creating lists of patients by diagnosis or health risk (eg, diabetes), by laboratory results (eg, patients with abnormal hematocrit levels), and by medications they currently take (eg, patients on warfarin), using a five-point scale: very easy, somewhat easy, somewhat difficult, very difficult, and cannot generate. Furthermore, physicians were asked if their practice had components of an EHR, specifically defined as “an integrated clinical information system that tracks patient health data, and may include such functions as visit notes, prescriptions, laboratory orders, etc” and were also surveyed on the availability and use of specific EHR components, such as structured problem or medication lists and electronic reporting and review of laboratory results. The survey instrument and study protocol were reviewed and approved by the Partners Healthcare Institutional Review Board (IRB). The instrument is available as an appendix to this article (See Multimedia Appendix 1).

### Analysis

We used SAS 9.1.3 (SAS Institute, Cary, NC), applying weights throughout our analysis to control for both our stratified sampling plan (which included over-sampling of specific groups) and for variable response rates in different strata (specialty, category of practice size, hospital affiliation, and urban/rural location). We used frequency weights (fweights) which are the inverse of the response proportion for each stratum or, equivalently, the weights were determined by taking the population size for each stratum divided by the number of responses. Conceptually, the fweight for a particular response corresponds to the number of physician practices in Massachusetts that this response represents. The ultimate purpose of this weighting strategy was to make our results representative of the population of ambulatory care physician practices in Massachusetts.

We used logistic regression to assess the relationship between the presence of electronic health records in the practice and the ability to create diagnosis registries, laboratory test registries, and medication registries, adjusting each model for the following potential confounding factors:

- urban/rural location
- practice size
- practice ownership (owner, part-owner, non-owner)
- teaching status (whether any students or residents were present in the past year)
- hospital affiliation
- practice type (chosen from solo primary care practice, solo specialty care practice, primary care group/partnership, single specialty group/partnership, multi-specialty group/partnership)

In a secondary analysis limited to practices that had EHRs, we used chi-square tests to examine the relationship between use of key EHR features and the ability to generate each type of registry (diagnosis, laboratory test, medication). For the feature-specific analysis we looked at the effect of problem list use on the ability to generate diagnosis registries, electronic laboratory result review on laboratory test registries, and electronic medication list use on medication registries. In each one of the three cases, the associated feature was chosen for analysis because it was the most directly related feature to the registry type.

## Results

A total of 1345 physicians (71%, 1345 of 1884) completed the survey, 1328 by mail and 17 by phone. There were no significant differences between respondents and non-respondents on the sampling characteristics (specialty, practice size, hospital affiliation, and rural practice). The practices reported using a wide variety of commercially available and self-developed EHR systems. Table 1 shows the practice characteristics. Note that some respondents omitted certain questions on the survey so the number of practices does not always add up to 1345. The rural/urban classification was applied based on practice location, so it was available for all practices.

Among the 356 practices which had an EHR and reported its name, a total of 187 (52.5%) used one of the 4 most prevalent systems while the remaining 169 (47.5%) reported using one of 78 other systems that were named. There were also 31 practices that reported having an EHR but did not provide its name—they were still counted as having an EHR for purposes of the analysis.

**Table 1:** Practice characteristics

		n of practices	% of practices
**Practice Location**		
	Rural	331	24.6
	Urban	1014	75.4
**Practice Size**		
	≥ 6 physicians	504	37.5
	3 - 5 physicians	280	20.8
	1 - 2 physicians	383	28.5
**Practice Ownership**		
	Full owner	460	34.2
	Part owner	172	12.8
	Non owner	542	40.3
**Practice Involvement in Teaching**		
	Involved in teaching	552	41.0
	Not involved in teaching	628	46.7
**Hospital Affiliation**		
	Hospital based practice	360	26.8
	Non-hospital based practice	974	72.4
**Practice Type**		
	Solo primary care practice	154	11.4
	Solo specialty care practice	192	14.3
	Primary care group/partnership	309	23.0
	Single specialty group/partnership	338	25.1
	Multi-specialty group/partnership	177	13.2
**EHR Usage**		
	Yes	387	28.8
	No	794	59.0

Overall, 79.8% of physicians reported that their practices could generate registries of patients with a particular diagnosis; 56.1% could generate registries of patients with a specific laboratory result; and 55.8% could generate registries of patients taking a particular medication. Among physicians who reported that their practices were able to generate registries, the reported ease with which such registries could be generated varied greatly, as shown in Table 2. While 38.9% of physician practices that could generate diagnosis registries said their practice could do it easily or very easily, considerably fewer said that it was easy or very easy to generate registries based on laboratory test results (14.5%) or medications (17.8%).

**Table 2:** Ease or difficulty of generating registries of patients based on diagnosis, laboratory result and medication use^a^

Ease or Difficulty	Diagnosis registry	Laboratory result registry	Medication registry
Very Easy	15.0%	6.5%	7.4%
Somewhat Easy	23.9%	8.0%	10.4%
Somewhat Difficult	21.7%	16.0%	14.1%
Very Difficult	19.2%	25.6%	23.9%
Cannot Generate	20.2%	43.9%	44.2%

^**a**^Weighted proportion of physicians reporting that their practice can generate each kind of registry with a particular ease or difficulty.

Table 3 shows the proportion of physician practices that were capable of carrying out registry functions, stratified by pre-specified subgroups of interest. For all three registry types, providers with EHRs were significantly more likely to be able to perform registry functions than providers using non-electronic record systems (*P* < .001 for all three registry types). Also, larger practices, practices involved in teaching, hospital-based practices, and primary care practices were more likely to be able to generate registries.

In logistic regression analyses controlling for urban/rural location, practice size, practice ownership, teaching status, hospital affiliation, and practice type, the relationship between the presence of EHR and the ability to carry out each registry function remained robust. EHR adopters were more likely than non-adopters to be able to develop registries based on diagnosis (adjusted odds ratio [OR] 1.53, 95% confidence interval [CI] 1.25 - 1.86), laboratory results (OR 1.42, 95% CI, 1.22 - 1.66), and medications (OR 2.30, 95% CI, 1.96 - 2.70). Rural location, practice size, practice ownership, hospital affiliation, and practice type also remained significant correlates of one or more registry capability in the multivariate analyses (Table 4).

**Table 3:** Ability to perform registry functions according to practice characteristics (these data are weighted but not adjusted for confounding factors)

		Percentage of physicians able to perform function		
		Diagnosis registry	Laboratory result registry	Medication registry
**Practice Location**^**a**^				
	Rural	78.9%	58.9%	62.6%
	Urban	79.9%	55.9%	55.3%
**Practice Size**^**b**^				
	≥ 6 physicians	82.4%	60.1%	59.2%
	3 - 5 physicians	84.8%	63.4%	53.9%
	1 - 2 physicians	75.8%	50.6%	54.8%
**Practice Ownership**^**c**^				
	Full owner	77.8%	51.2%	54.1%
	Part owner	81.6%	49.9%	48.3%
	Non owner	81.6%	66.6%	61.7%
**Practice Involvement in Teaching**^**d**^				
	Involved in teaching	85.0%	63.2%	61.8%
	Not involved in teaching	76.9%	52.3%	52.5%
**Hospital Affiliation**^**e**^				
	Hospital based practice	81.9%	69.9%	65.2%
	Non-hospital based practice	79.4%	54.5%	54.8%
**Practice Type**^**d**^				
	Solo primary care practice	74.2%	57.2%	61.7%
	Solo specialty care practice	77.1%	47.0%	55.8%
	Primary care group/partnership	88.6%	71.4%	67.2%
	Single specialty group/partnership	78.5%	56.0%	44.5%
	Multi-specialty group/partnership	84.5%	58.7%	59.7%
**EHR Usage**^**d**^				
	Yes	85.9%	66.7%	71.6%
	No	78.0%	52.9%	51.1%

^a^ *P* = .64 using a chi-square test for diagnosis registry functions, *P* = .25 for laboratory registry functions, *P* = .005 for medication registry functions.

^b^ *P* < .001 for diagnosis and laboratory registry functions, *P* = .009 for medication registry functions.

^c^ *P* = .004 for diagnosis registry functions and *P* < .001 for laboratory and medication registry functions.

^d^ *P* < .001 for all three registry types.

^e^ *P* = .153 for diagnosis registry functions, *P* < .001 for medication and laboratory registry functions.

**Table 4:** Multivariate correlates of registry function capability

		Diagnosis registry OR (95% CI)	Laboratory result registry OR (95% CI)	Medication registry OR (95% CI)
**Practice Location**				
	Rural	0.95 (0.73 - 1.23)	1.08 (0.86 - 1.34)	1.32 (1.05 - 1.65)
	Urban	1 (Ref)	1 (Ref)	1 (Ref)
**Practice Size**				
	≥ 6 physicians	1.09 (0.85 - 1.41)	0.92 (0.74 - 1.14)	1.10 (0.89 - 1.37)
	3 - 5 physicians	1.62 (1.29 - 2.03)	1.39 (1.16 - 1.68)	1.20 (1.00 - 1.44)
	1 - 2 physicians	1 (Ref)	1 (Ref)	1 (Ref)
**Practice Ownership**				
	Full owner	1.28 (1.04 - 1.58)	0.71 (0.60 - 0.85)	0.65 (0.54 - 0.78)
	Part owner	1.19 (0.92 - 1.53)	0.53 (0.43 - 0.65)	0.71 (0.58 - 0.87)
	Non owner	1 (Ref)	1 (Ref)	1 (Ref)
**Practice Involvement in Teaching**				
	Involved in teaching	1.42 (1.19 - 1.70)	0.98 (0.85 - 1.13)	1.08 (0.94 - 1.25)
	Not involved in teaching	1 (Ref)	1 (Ref)	1 (Ref)
**Hospital Affiliation**				
	Hospital based practice	1.09 (0.85 - 1.39)	1.87 (1.52 - 2.30)	1.35 (1.10 - 1.66)
	Non-hospital-based practice	1 (Ref)	1 (Ref)	1 (Ref)
**Practice Type**				
	Solo primary care practice	0.67 (0.47 - 0.96)	1.23 (0.92 - 1.65)	1.97 (1.46 - 2.66)
	Solo specialty care practice	0.84 (0.60 - 1.17)	0.90 (0.68 - 1.18)	1.78 (1.35 - 2.34)
	Primary care group/partnership	1.47 (1.07 - 2.02)	1.90 (1.50 - 2.42)	1.61 (1.26 - 2.04)
	Single specialty group/partnership	0.70 (0.54 - 0.91)	0.91 (0.74 - 1.13)	0.62 (0.50 - 0.77)
	Multi-specialty group/partnership	1 (Ref)	1 (Ref)	1 (Ref)
**EHR Usage**				
	Yes	1.53 (1.25 - 1.86)	1.42 (1.22 - 1.66)	2.30 (1.96 - 2.70)
	No	1 (Ref)	1 (Ref)	1 (Ref)

OR = adjusted odds ratio; CI = confidence interval

Ref: Reference Category

We also observed a relationship between use of key EHR features and ability to perform related registry functions. Specifically, within the group of physicians who had access to an EHR in their practice, 90.4% of physicians who reported using an electronic problem list at least some of the time had the ability to perform diagnosis registry functions, while only 67.7% of physicians using an EHR without access to an electronic problem list could perform these functions (*P* < .001). Similarly, while 75.2% of physicians who used an electronic medication list could perform medication registry functions, only 53.0% of physicians who used an EHR without a medication list reported they could perform them (*P* < .001). Finally, while 71.5% of physicians who used their EHR to view laboratory results could perform laboratory registry functions, only 33.9% of physicians whose EHR could not be used to view laboratory results reported they could perform these functions (*P* < .001).

## Discussion

### Principal Results

While many studies have demonstrated the value of being able to perform registry functions for improving the quality and safety of health care [2-3,6-13], few data are available regarding the capability of carrying out registry functions in community-based practices. In this study, about 80% of physicians reported being able to generate registries of patients according to diagnoses, but nearly half of all physicians in ambulatory care practice in Massachusetts could not create registries by medication or laboratory result.

Having EHRs was strongly associated with the reported ability to generate registries based on diagnosis, laboratory test result, or medication, but even among EHR users, 14% could not generate lists of diagnoses, 33% could not do so for laboratory tests, and 28% could not do so for medications. Furthermore, we found that physicians who reported active use of key EHR functions were considerably more likely to report being able to generate registries. Thus, these data suggest that EHRs appear important for delivering care using registries, and that most but not all EHR users could generate registries using their electronic records.

We are uncertain about why some physician practices with EHRs were unable to create registries. Many of these practices reported using EHRs which we knew to have this capability. It is likely that at least some of the EHR users who reported an inability to generate registries actually have the ability to generate them using their EHR, but are unaware of the feature. This suggests that improvements in documentation, training, and ease of use to help more physicians take advantage of the existing registry capabilities of their EHRs may be useful.

We were also a bit surprised by the relatively high proportion of EHR non-users who reported being able to generate registries. We are uncertain as to the mechanism employed by these users, since our survey did not ask them to explain how they were generating registries. These users may have been using retrospective chart review, prospective tracking, or analysis based on administrative data (such as billing and claims data in a practice management system). Each of these methods has a significant downside. Retrospective chart review is extremely time-consuming and error prone; prospective tracking requires criteria to be developed in advance; and non-clinical data are often less sensitive and specific than clinical data.

Taken together, our findings raise concerns about the ability of many ambulatory care practices, particularly the majority of practices without EHRs, to provide effective care for their patients on a population level. Physicians and practices need to consider population-level care management, not only as an essential component to effective practice within the Chronic Care Model [2,3], but also as a necessary tool for responding to the exigencies of forces driving quality improvement, such as pay-for-performance. Without EHRs, and without active and effective use of key features in robust EHRs [16], physicians and practices will have much greater difficulty in efficiently delivering safe and effective care for patients with acute and chronic problems.

EHRs can and should either include the inbuilt ability to query across patient records by a variety of criteria, or support extracting patient data which can be fed into other applications which do this. The ability to generate registries by diagnosis is common in many commercial EHR systems. In fact, it is a requirement of the 2007 and 2008 Certification Commission for Health Information Technology (CCHIT) criteria for ambulatory EHR certification [17]. However, such certification is voluntary, and many commercial products are not certified, or are certified under the older 2006 criteria, which did not require the availability of registry functions. CCHIT certification is valid for only three years, however, so as products certified under the 2006 criteria are re-certified under the current criteria, this gap will close. Also, although certification is voluntary, there are increasing incentives (or, in some limited cases, mandates) encouraging use of CCHIT-certified products, so the use of non-certified products which may not have registry capabilities is likely to be lessened over time [18]. Furthermore, just because an EHR can be used to generate a registry, the software does not always make it easy or user-friendly for a provider to do so. However, even when EHRs do not come “pre-loaded” with the ability to generate registries, the organization of patient information in structured fields in an electronic format facilitates the creation of registries more easily than in paper-based systems.

It is also worth noting that the ability to create registries in an EHR is generally predicated on the use of structured documentation features within the EHR. For example, if a clinician documents patient problems only in unstructured clinical notes, it is nearly impossible in most commercially available EHRs to build medication registries based on this unstructured information. However, if the clinician uses a structured medication list with a controlled medication vocabulary, generating such a registry becomes much easier.

Finally, our survey was conducted in 2005. Since then, adoption of EHRs in ambulatory practices has increased somewhat [19], though is still far from universal. Since our data suggest that physicians with EHRs are more able to generate registries than non-users, we expect that the current ability of physicians to generate such registries is likely now higher than it was in 2005. Moreover, the capabilities of EHR systems, as a rule, increase over time, so we likewise expect that EHR users are more likely, today, to be able to generate registries than they were in 2005.

### Implications

Our findings have several important implications for physicians, for the health care system, and for developers of electronic health records. Because our findings suggest that the ability to generate registries is less than universal, and because generating registries is integral to quality and safety enhancing activities, it may be necessary to take steps to increase these capabilities in office practice. Providing physicians and practices with training and activities to increase awareness of the role of generating registries may be beneficial, but these changes are unlikely to be sufficient. Incentives also likely play an important role; physicians are more likely to adopt and use registry functions if financial incentives are in place to do so [20]. As pay-for-performance initiatives become more prevalent, physicians are likely to embrace the use of registries as a foundation for building practice-level population management capabilities. Our findings also show that EHR users are more likely to be able to generate registries than non-users, so incentives aimed at EHR adoption alone are also likely to have a positive effect on registry capabilities.

### Limitations

The study has several limitations. First, our survey was limited to physicians in ambulatory care in Massachusetts, and the results may not be generalizable to other states or regions. However, given that Massachusetts is a state in which more than 45% of physicians have EHRs [15], the large proportions of physicians and practices reporting inability to perform registry functions in this study are likely to be even larger in other states, where EHR adoption has lagged. As such, the need to consider efforts to adopt EHRs and expand their use may have even greater imperative in other regions.

Another limitation is the self-reported nature of survey studies such as ours. We are, of course, not truly measuring physicians’ abilities to generate registries, but instead their self-reports of the ability to generate three specific types of registries. This raises the possibility of social desirability bias influencing physicians’ responses to survey questions. However, if this bias were present, then one might expect that physicians overestimated their abilities to perform registry functions, which would mean that even fewer physicians than reported have the ability to generate registries. Also, our survey asked providers how easily they could perform registry functions but did not ask how frequently they actually did perform such functions, or for what purposes and with what results. It is important to note that, among those practices that reported the ability to make these registries, we do not know the frequency with which they did so. It is possible that some practices, although able to create the registries, never actually do. Future qualitative and quantitative studies should explore how physicians and practices are using registries, as well as the barriers to, and facilitators of, effective use of these important tools. Intervention studies will then be able to test strategies for improving physicians’ use of registries to improve quality of care and patient safety.

Finally, our survey was limited to registries of diagnoses, medications and lab results. Other types of registries exist, such as registries of patients receiving a particular surgical procedure, which are often used for tracking quality and outcomes, tumor registries, and registries of implanted devices, such as implantable cardiac defibrillators or pacemakers, which are important in the event of a recall. Such registries are generally used in specific specialties, and it would be worthwhile to survey specialists about their use of these special-purpose registries.

### Conclusion

While registry functions are available to many physicians, their availability is far from universal. Because generating registries is essential for population health management activities associated with improved quality, safety, and efficiency, it is important that their availability increase. Adoption of EHRs appears to be a useful step toward this end, since practices with EHRs are significantly more likely to be able to carry out registry functions. CCHIT should intensify and expand its requirements for registry function capabilities, and commercial EHR products without these capabilities should be extended to provide them. Health policy makers and health care leaders can then develop and disseminate strategies for using registries for improving patient safety and the quality of health care.

## Multimedia Appendix 1

Massachusetts Survey of Physicians and Computer Technology

## Abbreviations

CCHIT: Certification Commission for Health Information Technology
EHR: electronic health record
IRB: institutional review board
