# Supplementary material for: Ability to Generate Patient Registries Among Practices With and Without Electronic Health Records
Source: J Med Internet Res. 2009 Aug 10;11(3):e31. doi: 10.2196/jmir.1166 (PMC2762852; doi:10.2196/jmir.1166)
Supplement: Supplementary file 1 [file jmir_v11i3e31_app1.pdf]

Massachusetts Survey of Physicians and Computer Technology

Instructions

This survey asks about your medical practice and factors related to the use of certain computer technology, particularly electronic health records (EHRs; also called electronic medical records). It will take about 15 minutes to complete.

All responses are private and confidential. Results will be analyzed only in the aggregate and individual responses will not be reported.

Section I. Practice Characteristics
In this section, we ask you questions about your outpatient practice.

1. In a typical week, in how many different outpatient offices do you see patients?

- ❑1 One
- ❑2 Two
- ❑3 Three or more
- ❑4 None, I do not see any outpatients (***SKIP*** *to Question 30 on Page 7*)

For the remainder of the survey, please keep in mind the office practice site where you spend the most time, your ***main*** *practice.*

---

2. What percent of your **outpatient** clinical time is spent at your (main) practice?

- ❑1 Less than 25% of outpatient time
- ❑2 25%-49% of outpatient time
- ❑3 50% - 75% of outpatient time
- ❑4 More than 75% of outpatient time

3. How would you best characterize your practice? *(Please check only* ***one****)*

- ❑1 Solo primary care practice
- ❑2 Solo specialty care practice
- ❑3 Primary care group or partnership
- ❑4 Single specialty group or partnership
- ❑5 Multi-specialty group or partnership (including staff or group model HMOs)

4. How long have you been associated with your practice group?

___ ___ years

5. Are you a

- ❑1 Full-owner
- ❑2 Part-owner
- ❑3 Not an owner of the practice

NOT FOR CITATION OR REPRODUCTION

6. Considering all full- and part-time clinicians at your main practice, including yourself, how many are

   Physicians: ___ ___ ___

   Nurse practitioners or physician assistants: ___ ___ ___

7. Have any residents or students been present in your practice within the past year?

   ❑1 Yes
   ❑2 No

8. Please **estimate** the number of outpatient visits **you** have in a **typical week** in your practice.

   ___ ___ ___ outpatient visits

9. Please **estimate** approximately what percentage of the **patients you see** in a **typical week** are of each race/ethnicity:

a)	Asian	___ ___ %	should total 100%
b)	American Indian or Alaska Native	___ ___ %	
c)	Black or African American, non-Hispanic	___ ___ %	
d)	Native Hawaiian or Other Pacific Islander	___ ___ %	
e)	White, non-Hispanic	___ ___ %	
f)	Hispanic or Latino	___ ___ %	
g)	Other	___ ___ %	

10. With your current medical record system (paper and/or electronic), how easy would it be for you or your staff to generate the following information about your patients?

		Very Easy	Somewhat Easy	Somewhat difficult	Very Difficult	Cannot Generate
a)	List of patients by diagnosis or health risk (e.g., diabetes)	❑1	❑2	❑3	❑4	❑5
b)	List of patients by laboratory results (e.g., patients with abnormal hematocrit levels)	❑1	❑2	❑3	❑4	❑5
c)	List of patients by medications they currently take (e.g., patients on warfarin)	❑1	❑2	❑3	❑4	❑5

11. Thinking of your main practice, please indicate how much of a problem each of the following is for you: *(Check only **one** for each item.)*

		Not a Problem	Slight Problem	Moderate Problem	Serious Problem
a)	Isolation from colleagues	❑1	❑2	❑3	❑4
b)	Personal or professional stress	❑1	❑2	❑3	❑4
c)	Having to work long hours to meet practice demands	❑1	❑2	❑3	❑4
d)	Feeling demoralized about the state of medical practice in general	❑1	❑2	❑3	❑4

NOT FOR CITATION OR REPRODUCTION

12. Overall, how satisfied are you with your current practice situation?

- ❑1 Very satisfied
- ❑2 Generally satisfied
- ❑3 Somewhat dissatisfied
- ❑4 Very dissatisfied

<u>Section II: Health Information Technology</u>

The next set of questions will ask you about the computers and health information technology in your main office practice. Please select the answer that best describes your practice.

13. Does your practice use a **computerized scheduling system**?

- ❑1 Yes → (*If "Yes", please* ***answer a.***)
  a. For how many years has your practice used a computerized scheduling system?
  ___ ___ years
- ❑2 No

14. Upon completing a typical office visit, how do you generate medication prescriptions?

- ❑1 Computerized, with decision support (e.g., drug interaction alerts)
- ❑2 Computerized, no decision support
- ❑3 Handwritten
- ❑4 Other (Describe:____)

15. Does your main practice have components of any electronic health record (EHR), that is, an integrated clinical information system that tracks patient health data, and may include such functions as visit notes, prescriptions, lab orders, etc?

- ❑1 Yes
- ❑2 No → (*If "No", please* ***answer a.*** *and then* ***SKIP to Question 19***)
  a. When do you plan to implement an EHR?
  - ❑1 Within the next 12 months
  - ❑2 Within the next 1-2 years
  - ❑3 Within the next 3-5 years
  - ❑4 No specific plans

16. What is the name of your EHR system (e.g. Epic, Logician): ___________________________

17. Please indicate when your practice **first** began using an EHR:

___ ___ / ___ ___ ___ ___ (month / year)

NOT FOR CITATION OR REPRODUCTION

18. Please indicate all **features of the EHR** that you **have available** in your practice. For those features that you have, indicate the extent to which **you use** them:

	**Available**			**Use**		
**Features of your EHR**	Yes	No	Don't Know	I do not use	I use *some* of the time	I use *most or all* of the time
a) Laboratory test results	❑1	❑2	❑3	❑1	❑2	❑3
b) Laboratory order entry	❑1	❑2	❑3	❑1	❑2	❑3
c) Radiology test results	❑1	❑2	❑3	❑1	❑2	❑3
d) Radiology order entry	❑1	❑2	❑3	❑1	❑2	❑3
e) Electronic visit notes	❑1	❑2	❑3	❑1	❑2	❑3
f) Reminders for care activities (e.g. overdue health maintenance)	❑1	❑2	❑3	❑1	❑2	❑3
g) Electronic medication lists of what each patient takes	❑1	❑2	❑3	❑1	❑2	❑3
h) Electronic problem list	❑1	❑2	❑3	❑1	❑2	❑3
i) Can transmit prescriptions to pharmacy electronically or via electronic faxing	❑1	❑2	❑3	❑1	❑2	❑3
j) Electronic referrals or clinical messaging (secure e-mailing between providers)	❑1	❑2	❑3	❑1	❑2	❑3

19. How much of a barrier is each of the following to beginning or expanding the use of computer technology in your main practice?

	Not a barrier	Minor barrier	Major barrier
a) Computer skills of you and/or colleagues/staff	❑1	❑2	❑3
b) Computer technical support	❑1	❑2	❑3
c) Lack of time to acquire knowledge about systems	❑1	❑2	❑3
d) Start-up financial costs	❑1	❑2	❑3
e) Ongoing financial costs	❑1	❑2	❑3
f) Training and productivity loss	❑1	❑2	❑3
g) Physician skepticism	❑1	❑2	❑3
h) Privacy or security concerns	❑1	❑2	❑3
i) Lack of uniform standards within industry (e.g., having to use multiple systems used by different providers and plans)	❑1	❑2	❑3
j) Technical limitations of systems	❑1	❑2	❑3

NOT FOR CITATION OR REPRODUCTION

20. How much of a role do/did each of the following organizations play in deciding whether to adopt a new electronic health record system in your practice?

	Very much	Some what	Very little	Not at all	N/A or don't know
a) Your practice group(s)	❑1	❑2	❑3	❑4	❑5
b) Physician Hospital Organization(s) (PHOs) or Independent Practice Association(s) (IPAs)	❑1	❑2	❑3	❑4	❑5
c) Integrated Delivery System(s) (IDS) (e.g. Baystate, Partners)	❑1	❑2	❑3	❑4	❑5
d) Managed care plans you work with	❑1	❑2	❑3	❑4	❑5
e) Massachusetts Medical Society	❑1	❑2	❑3	❑4	❑5
f) Your specialty's professional society (e.g., AAP, AAFP, ACP, ACS, etc.)	❑1	❑2	❑3	❑4	❑5
g) MassPRO or DOQ-IT	❑1	❑2	❑3	❑4	❑5
h) Massachusetts e-Health Collaborative	❑1	❑2	❑3	❑4	❑5
i) The LeapFrog Group	❑1	❑2	❑3	❑4	❑5
j) Other (i. specify:_________________)	❑1	❑2	❑3	❑4	❑5

Section III. Personal Computer Experience

21. How often do you use the Internet for personal and/or professional use, including e-mail from home, work, or another location? *(Please check only **one**)*

- ❑1 Several times a day
- ❑2 Daily
- ❑3 Weekly
- ❑4 Monthly
- ❑5 Less than monthly or not at all

22. What type of Internet connection do you have at your main practice? *(Please check only **one**)*

- ❑1 Do not have an Internet connection at work
- ❑2 Dial-up modem connection
- ❑3 Broadband (i.e., DSL or cable modem) or faster connection (e.g. T1 or T3 line)
- ❑4 Don't know

23. Does your practice have email?

- ❑1 Yes
- ❑2 No

NOT FOR CITATION OR REPRODUCTION

Section IV: Computers and Health Care

24. For each outcome listed below, indicate whether you think the effect of computers is, or would be, very positive, somewhat positive, no effect, somewhat negative, or very negative:

Effect of computers on…	Very Positive	Somewhat Positive	No Effect	Somewhat Negative	Very Negative
a) Controlling costs of health care	❑1	❑2	❑3	❑4	❑5
b) Quality of health care	❑1	❑2	❑3	❑4	❑5
c) Interactions within the health care team	❑1	❑2	❑3	❑4	❑5
d) Patient-physician communication	❑1	❑2	❑3	❑4	❑5
e) Patient privacy	❑1	❑2	❑3	❑4	❑5
f) Clinicians' access to up-to-date knowledge	❑1	❑2	❑3	❑4	❑5
g) Efficiency of providing care	❑1	❑2	❑3	❑4	❑5
h) Medication errors	❑1	❑2	❑3	❑4	❑5

Section V: Financial Considerations

25. Please indicate below whether the following factors (through bonuses, returned withholds, or other incentive payments) contribute to **either** your **practice's income**, or **your personal earnings**?

	**Practice's Income**		**Personal Earnings**	
	Yes	No	Yes	No
a) Types of electronic information systems you **have** (e.g., EHRs, e-prescribing)	❑1	❑2	❑1	❑2
b) The amount you **use** electronic information systems	❑1	❑2	❑1	❑2
c) Patient survey results (e.g. satisfaction)	❑1	❑2	❑1	❑2
d) Clinical quality (e.g., "pay for performance")	❑1	❑2	❑1	❑2

26. Approximately what percent of your 2004 **clinical practice income** was earned in the form of bonuses, returned withholds, or other incentive payments **based on the use of electronic health record systems or electronic prescribing**?

❑1 0% of income
❑2 1 - 5% of income
❑3 6 - 10% of income
❑4 more than 10% of income
❑5 Not sure

27. Practices vary with respect to the capital they have available for expansion and improvement. What financial resources does your main practice have for expansion or improvements of any kind?

❑1 Extensive resources
❑2 Moderate resources
❑3 Limited resources
❑4 No resources

28. If you decided that a new computer system would improve health care quality and was worth the financial investment, how difficult would it be for your practice to purchase such a system if the cost was…

	Not at all Difficult	Somewhat Difficult	Very Difficult	Impossible
a) Less than $10,000 per physician	❑1	❑2	❑3	❑4
b) $10,000 - $25,000 per physician	❑1	❑2	❑3	❑4
c) Greater than $25,000 per physician	❑1	❑2	❑3	❑4

Section VI: The Office Practice Environment

29. Please indicate your agreement or disagreement with the following statements, considering your main office practice:

	Strongly Agree	Agree	Neither Agree nor Disagree	Disagree	Strongly Disagree
a) The office staff are innovative	❑1	❑2	❑3	❑4	❑5
b) The physician(s) are innovative	❑1	❑2	❑3	❑4	❑5
c) Among my colleagues, I am usually one of the first to find out about a new diagnostic test or treatment	❑1	❑2	❑3	❑4	❑5
d) We are actively doing things to improve quality of care	❑1	❑2	❑3	❑4	❑5
e) After we make changes to improve quality, we evaluate their effectiveness	❑1	❑2	❑3	❑4	❑5
f) We have quality problems in our practice	❑1	❑2	❑3	❑4	❑5
g) Our procedures and systems are good at preventing errors from occurring	❑1	❑2	❑3	❑4	❑5

Section VII: Personal Characteristics

We would like to end this survey by asking about general background information that may help us interpret survey findings and determine how representative our sample is.

30. In what year did you graduate from medical school?

___ ___ ___ ___ year graduated

31. In what year were you born?

___ ___ ___ ___ year

32. Are you

❑1 Male
❑2 Female

NOT FOR CITATION OR REPRODUCTION

33. Are you of Hispanic or Latino origin?

- ❑1 Yes
- ❑2 No

34. Please answer this question whether or not you are Hispanic or Latino. What is your race? **Select one or more** of the following -

- ❑1 Asian
- ❑2 American Indian or Alaska Native
- ❑3 Black or African American
- ❑4 Native Hawaiian or Other Pacific Islander
- ❑5 White
- ❑5 Other

35. Date survey completed: ___ ___ / ___ ___ / ___ ___ ___ ___
Month Day Year

Please return the survey in the stamped return envelope to **(Atlantic to insert):**

Thank you for your help!

NOT FOR CITATION OR REPRODUCTION
